# Enhanced Photocatalytic Degradation of Tetracycline and Oxytetracycline Antibiotics by BiVO_4_ Photocatalyst under Visible Light and Solar Light Irradiation

**DOI:** 10.3390/antibiotics11060761

**Published:** 2022-06-02

**Authors:** Khuanjit Hemavibool, Theepakorn Sansenya, Suwat Nanan

**Affiliations:** 1 Department of Chemistry, Faculty of Science, Naresuan University, Phitsanulok 65000, Thailand; khuanjitb@nu.ac.th; 2 Materials Chemistry Research Center, Center of Excellence for Innovation in Chemistry (PERCH-CIC), Department of Chemistry, Faculty of Science, Khon Kaen University, Khon Kaen 40002, Thailand; theepakorn_s@kkumail.com

**Keywords:** bismuth vanadate, removal, antibiotics, sunlight, tetracycline

## Abstract

The efficient degradation of a toxic antibiotic from an aqueous solution is essential for environmental protection. Our research aimed to fabricate a bismuth vanadate (BiVO_4_) catalyst via a facile hydrothermal method. The prepared catalyst exhibited a monoclinic phase with a band gap energy of 2.33 eV, indicating the excellent visible-light-active properties of a semiconductor. The photocatalytic performance of the synthesized BiVO_4_ catalyst was studied by determining the removal of tetracycline (TC) and oxytetracycline (OTC) antibiotics. After 240 min, under sunlight conditions, a high performance of 72% and 83% degradation of TC and OTC, respectively, was achieved. The photocatalytic degradation of the antibiotics correlates well with a first-order reaction, with a high rate constant of 0.0102 min^−1^. Photogenerated electrons and holes played an important role in the removal of the pollutant. After photocatalytic study, the structural stability of the prepared bismuth vanadate photocatalyst was confirmed. The photocatalyst provided a promising performance even after five successive runs. The result indicates the excellent cycling ability of the sample. The present work demonstrates a promising route for the preparation of a BiVO_4_ catalyst for the complete removal of toxic antibiotics in aqueous solutions.

## 1. Introduction

Water pollution is widely accepted as an increasingly serious environmental hazard. Therefore, there is a tremendous attempt to remove organic pollutants that contaminate natural water resources [1,2,3]. Oxytetracycline (OTC) and tetracycline (TC) are the typical antibiotics widely used in human and veterinary medicine. In addition, these drugs were also used in agriculture, aquaculture, and other fields [4,5,6,7,8,9]. However, these drugs cause a serious problem to the environment [4,5,6,7,8,9]. Therefore, the complete removal of these antibiotics from aqueous phases has become a major aim. 

Various treatment methods have been used for the incomplete degradation of pollutants in wastewater. In some cases, however, the creation of secondary toxic organic pollutants was detected [10]. Alternatively, an advanced oxidation process based on semiconducting photocatalysis provides an alternative route for the complete degradation of toxic organic pollutants [11,12,13]. In general, it is known that the commercially available TiO_2_ photocatalyst is active under UV light. This photocatalyst, however, shows low photoactivity under natural sunlight. Interestingly, visible-light-active catalysts have gained much attention due to the advantages of utilizing abundant solar light [3,14,15,16,17].

Visible-light-responsive photocatalysts based on bismuth, such as bismuth molybdate, bismuth vanadate, and bismuth oxyhalides, have been fabricated [18,19,20,21,22,23,24]. Interestingly, the bismuth vanadate (BiVO_4_) catalyst has gained much attention. This catalyst can be categorized into three crystal phases: tetragonal, monoclinic, and tetragonal zircon (z-t). It has been shown that BiVO_4_ with a monoclinic crystal structure provides maximum photoactivity in comparison to tetragonal and tetragonal zircon crystal structures. Moreover, the BiVO_4_ photocatalyst with an Aurivillius structure has the advantages of low toxicity, good structural stability, and high visible-light-active performance [17,18]. Furthermore, this catalyst is cheap and stable against photo-corrosion. Both physical and chemical methods have been reported for the fabrication of BiVO_4_ [21,22,25,26,27,28]. It is well accepted that the hydrothermal/solvothermal route provides the benefits of being less complicated, low cost, and providing an excellent yield, with a high potential for large-scale preparation [20]. 

In this work, the hydrothermal growth of BiVO_4_ was demonstrated. The photocatalytic performance of the synthesized photocatalyst was examined by studying the degradation of tetracycline (TC) and oxytetracycline (OTC) antibiotics. The enhanced sunlight-active performance of about 72% and 93% degradation of TC and OTC antibiotics, respectively, was obtained. This promising performance indicates the excellent environmental remediation property of a synthesized BiVO_4_ catalyst for the detoxification of harmful pollutants in aqueous solutions. 

## 2. Experiment

### 2.1. Chemicals and Reagents

All chemicals were used without further purification. The ultrapure water (DI, 18.2 MΩ·cm^−1^) was used. 

### 2.2. Hydrothermal Syntesis of BiVO_4_

The BiVO_4_ catalyst was hydrothermally synthesized by using an autoclave. Firstly, solution A, based on dissolving 3.3954 g of Bi(NO_3_)_3_ 5H_2_O in 30 mL of 1.5 M HNO_3,_ was prepared. Secondly, solution B, based on dissolving 0.8189 g of NH_4_VO_3_ in 30 mL of 1.5 M NaOH, was prepared. Thirdly, solution B was added to solution A. Afterwards a yellow color and precipitation were detected. Finally, the mixture was transferred into a 100 mL Teflon-lined autoclave. A temperature of 180 °C and a time of 15 h were selected. After that, the precipitate was collected, washed with water and ethanol, and then dried at 60 °C for 12 h.

### 2.3. Characterization

The sample was characterized using the same procedures which can be found elsewhere [3,11,12,13]. The crystal structure of the sample was elucidated by a powder X-ray diffraction method using monochromatic Cu Kα radiation. The vibrational spectrum was monitored using a FT-IR spectrophotometer. Preparation of the sample was performed by using KBr pellets. The morphological structure and the elemental composition of the sample were elucidated by scanning electron microscopy and transmission electron microscopy. The oxidation states of all elements in the sample were studied by X-ray photoelectron spectroscopy (XPS) at BL 5.3, SLRI, Nakhon Ratchasima, Thailand. An ULVA-PHI 500 VersaProbe II with monochromatic Al Kα radiation was used as an excitation source. A C 1S peak at 284.6 eV was applied as a reference peak to calibrate the binding energy. 

### 2.4. Photodegradation Study

#### 2.4.1. Photocatalytic Degradation of the Antibiotics

The photocatalytic performance of the photocatalyst was investigated by studying the degradation of tetracycline (TC) and oxytetracycline hydrochloride (OTC) antibiotics under the simulated visible light (a Panasonic daylight lamp, 15 W) and natural solar light irradiation. The details of the photocatalytic study were reported previously [3,11,12,13].

The blank experiment was performed by illuminating the antibiotic solution without the addition of the BiVO_4_ photocatalyst. This confirmed the low contribution in the photolysis of the antibiotic. In addition, the control experiment, based on the addition of the photocatalyst to the antibiotic solution without light, was also performed. This dark condition provided data based on the contribution of the adsorption process. The photodegradation study was performed in an aqueous solution of either the TC or OTC antibiotic (concentration of 10 ppm and total volume of 200 cm^3^). We used 50 mg of BiVO_4_ catalyst. A solution of 5 cm^3^ was sampled after photo illumination. The exact content of TC and OTC antibiotics was examined by a spectrophotometric method. The λ_max_ of 358 and 368 nm were used for TC and OTC, respectively.

The performance of the antibiotic removal was calculated by Equation (1):

Performance (%) = (1 − C/C_0_) × 100% (1)

where C_0_ and C represent the initial concentration and the concentration of the pollutant solution after different time periods of photo illumination, respectively.

The photoactivity of the prepared BiVO_4_ was also examined by determining the photocatalytic degradation rate as follows:

dC/dt = −k_1_C (2)

ln(C_0_/C) = k_1_t (3)

where k_1_ is the rate constant of the reaction.

#### 2.4.2. Study of the Photocatalytic Degradation Mechanism

To study the major species that play an important role in the degradation of the antibiotics, additions of *t*-butanol, NaN_3_, EDTA-2Na, and K_2_Cr_2_O_7_ to quench hydroxyl radicals, superoxide anion radicals, holes, and electrons, respectively, were performed. Additionally, KI was used as a quencher of surface hydroxyl radicals and holes. About 5 mM of each quencher was incorporated into the presence of the catalyst (50 mg). 

To detect the hydroxyl radicals, the dispersion of the catalyst in a terephthalic acid solution (TA) was performed. The creation of these radicals was investigated by using a spectrofluorometric method (λ_excitation_ of 315 nm).

#### 2.4.3. Cycling Ability of the Photocatalyst

To confirm the cycling ability of the prepared catalyst, after the first round of photocatalytic study, the catalyst was filtered. It was then washed (server times) with ethanol and water. The catalyst was dried and then used in the second run. This reuse was performed for five cycles.

## 3. Results and Discussion

### 3.1. Characterization of the BiVO_4_ Catalyst

**Figure 1:**
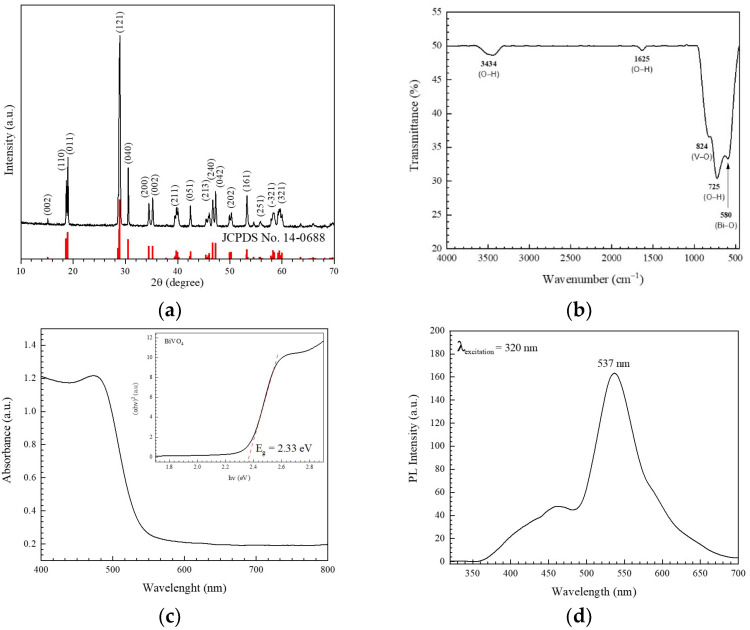
XRD pattern (**a**), FT-IR (**b**), UV-Vis (**c**), and PL spectrum (**d**) of the prepared BiVO_4_ catalyst.

The XRD pattern of the sample in Figure 1a displays the monoclinic scheelite phase of BiVO_4_ at 2θ of about 15.17, 18.64, 19.04, 28.93, 30.47, 34.51, 35.36, 39.88, 42.56, 46.09, 46.70, 47.26, 50.39, 53.37, 55.90, 58.35, and 59.81°, belonging to the reflection from the plane (002), (110), (011), (121), (040), (200), (002), (211), (051), (213), (240), (042), (202), (161), (251), (-321), and (321), respectively. These results corelate well with those reported in the literature, under the JCPDS No. 14-0688 file [29]. 

Figure 1b shows the FT-IR spectrum of the sample. The vibrational bands at 725 cm^−1^ and 824 cm^−1^ indicated the *ν*_3_ asymmetric and *ν*_1_ symmetric stretching vibration of VO_4_^3^, respectively. The Bi–O bond was confirmed by the existence of the vibrational peak at 580 cm^−1^ [29]. 

Figure 1c shows the UV–Vis diffuse reflectance spectrum of the BiVO4 catalyst with a band gap energy (E_g_) of 2.33 eV, calculated by the Kubelka-Munk formula [24,30,31,32]. Accordingly, a visible-light-active absorption edge of 532 nm was obtained. Furthermore, the electron-hole recombination rate of the sample was studied by monitoring the photoluminescence (PL) spectrum, as shown in Figure 1d. The spectrum showed a peak at about 537 nm, corresponding to near band edge (NBE) emission, which is normally found in a sample with high crystallinity [20].

**Figure 2:**
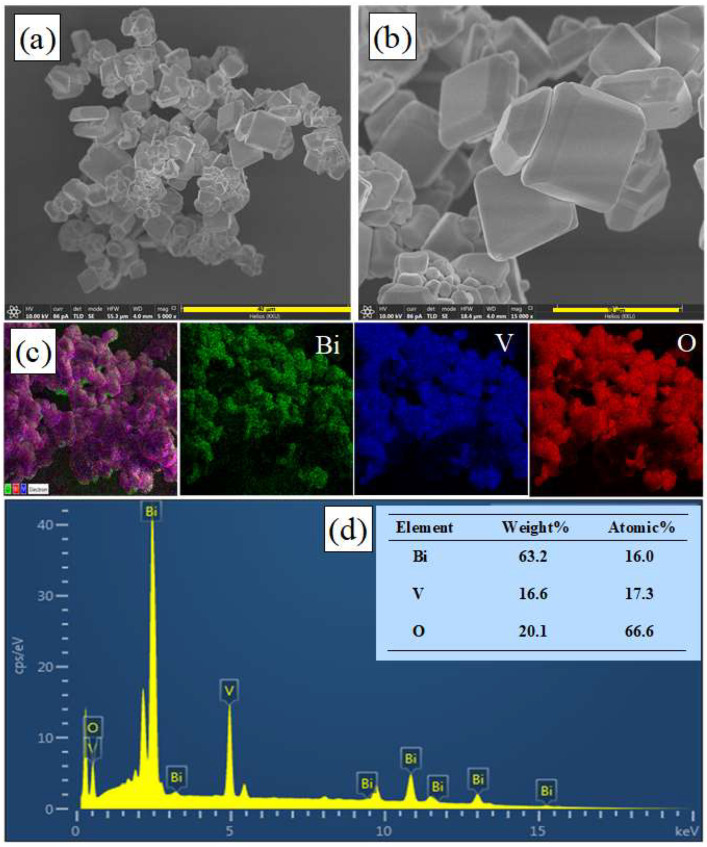
SEM micrographs (**a**,**b**), SEM micrograph of the mapping area and EDX elementary mapping of Bi, V, and O (**c**), and EDX spectrum (**d**) of BiVO_4_.

Figure 2a,b show the SEM micrographs of BiVO_4_ with cubic microstructures of 5.6–9.0 μm. The prepared catalyst showed the particles to have a well-faceted structure and high surface quality [3]. The SEM images of the mapping area and elemental color mapping (Figure 2c) revealed a uniform dispersion of Bi, V, and O elements in the prepared catalyst. The elemental compositions of the as-prepared sample were examined by energy dispersive X-ray spectroscopy (EDX). The weight percentages of Bi:V:O were 63.2:16.6:20.1, while the corresponding atomic percentages were 16.0:17.3:66.6. All in all, the results support a Bi:V:O ratio of 1:1:4 within the catalyst (Figure 2d). 

**Figure 3:**
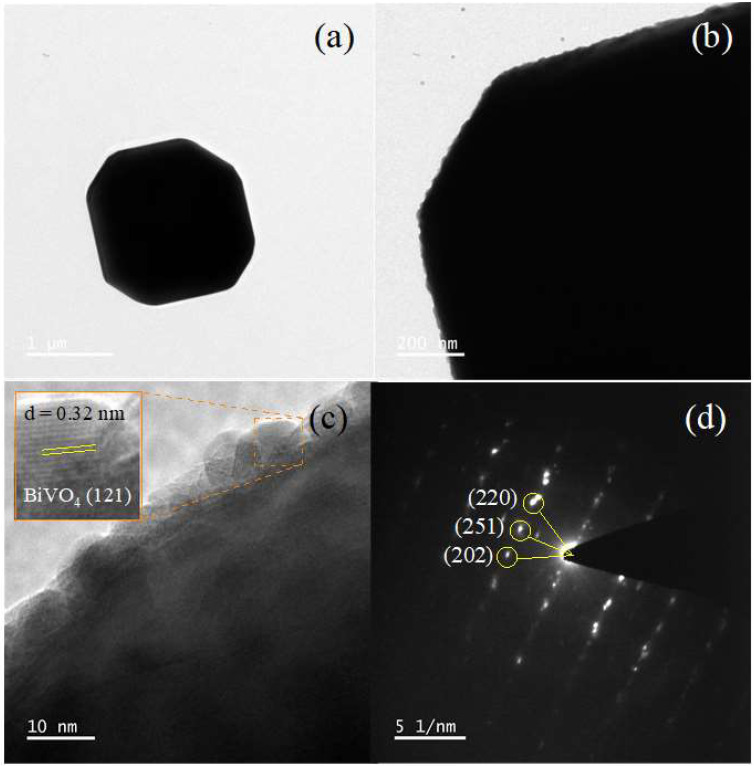
TEM micrographs (**a**,**b**), HR-TEM micrograph (**c**), and SAED pattern (**d**) obtained from the prepared BiVO_4_ catalyst.

In addition, TEM and SAED methods were carried out to examine the structural information of the prepared BiVO_4_ catalyst (Figure 3). The TEM images of the catalyst in Figure 3a,b display a size of about 5.28 μm. In addition, the crystalline structure was obtained from the high-resolution TEM (HR-TEM) micrograph (Figure 3c). On examination of the lattice fringes, the interplanar lattice spacing (d) of 0.32 nm is due to the reflection from the (121) crystal plane of the monoclinic BiVO_4_. The selected area electron diffraction (SAED) pattern in Figure 3d revealed the monocrystalline nature of the catalyst [3,33]. The SAED pattern was mainly caused by the reflection from the (202), (220), and (251) crystal planes. This is in good agreement with those found in the XRD diffractogram results (Figure 1a).

**Figure 4:**
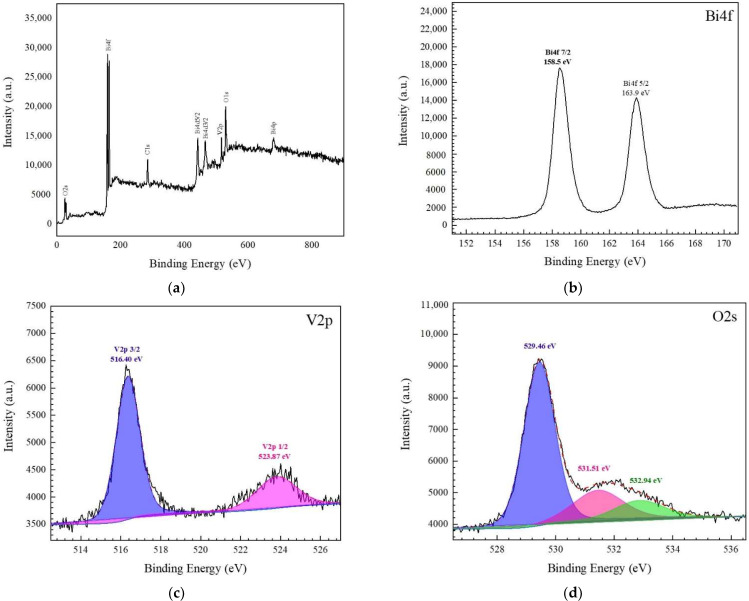
XPS survey scan (**a**), core level spectra of Bi 4f (**b**), V 2p (**c**), and O 1s (**d**) of the prepared BiVO_4_ catalyst.

The surface elemental composition and chemical oxidation state of the prepared catalyst was examined by an XPS technique. The correction of binding energies was carried out by using a peak of carbon (C) 1s at 284.6 eV as a reference. The XPS survey spectrum (Figure 4a) confirmed the existence of Bi, V, and O elements, correlating with the results from the XRD and EDS. Figure 4b shows the XPS spectrum of Bi 4f with two XPS peaks at 163.9 and 158.5 eV, assigned to those of Bi 4f_5/2_ and Bi 4f_7/2_, respectively. The result confirmed the presence of Bi^3+^. In the case of the vanadium element (V 2p), the two peaks at 523.87 and 516.40 eV, as seen in Figure 4c, were assigned to V 2p1/2 and V 2p3/2, respectively. Moreover, in the case of the O 1s spectrum (Figure 4d), the deconvolution of the spectrum ends up with three XPS peaks at 532.94, 531.51, and 529.46 eV; this can be attributed to the O 1s from the adsorbed water on the surface, the hydroxyl group, and the lattice oxygen in the catalyst, respectively [3,34]. 

**Figure 5:**
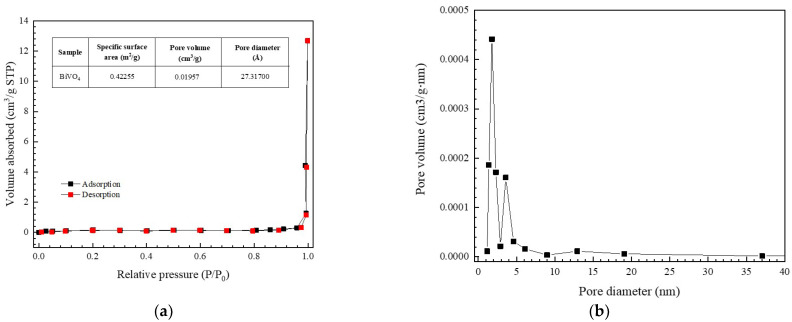
Nitrogen adsorption-desorption isotherm (**a**) and the pore size distribution (**b**) of BiVO_4_.

The textural properties of the BiVO_4_ catalyst were examined from a nitrogen (N_2_) adsorption-desorption isotherm. According to the IUPAC classification, the BiVO_4_ catalyst showed type IV isotherm properties. In addition, a distinct H_3_ hysteresis loop was detected at a high relative pressure (Figure 5a) [3,35]. The catalyst showed a mesoporous structure with a confirmed specific surface area of 0.42 m^2^/g. In addition, a Barett-Joyner-Halenda (BJH) pore volume of about 0.020 cm^3^/g and an average pore diameter of about 27.31 nm were observed. 

An electrochemical method was used to study the spatial transfer and separation of electron-hole pairs. Firstly, linear sweep voltammetry (LSV) was investigated. As seen in 6a, the photocurrent density of BiVO_4_ under photo illumination was greater than that obtained under the dark condition. We conclude that, upon the light irradiation of the prepared catalyst, the enhancement of its photogenerated carrier ability is produced. This results in an improvement in the photoactivity of the synthesized photocatalyst [3]. 

**Figure 6:**
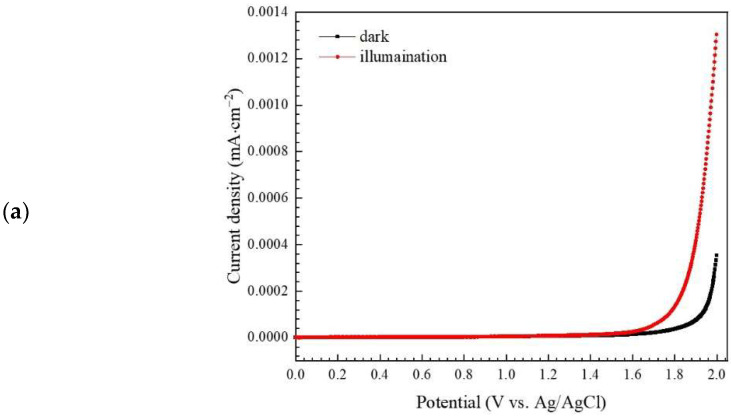

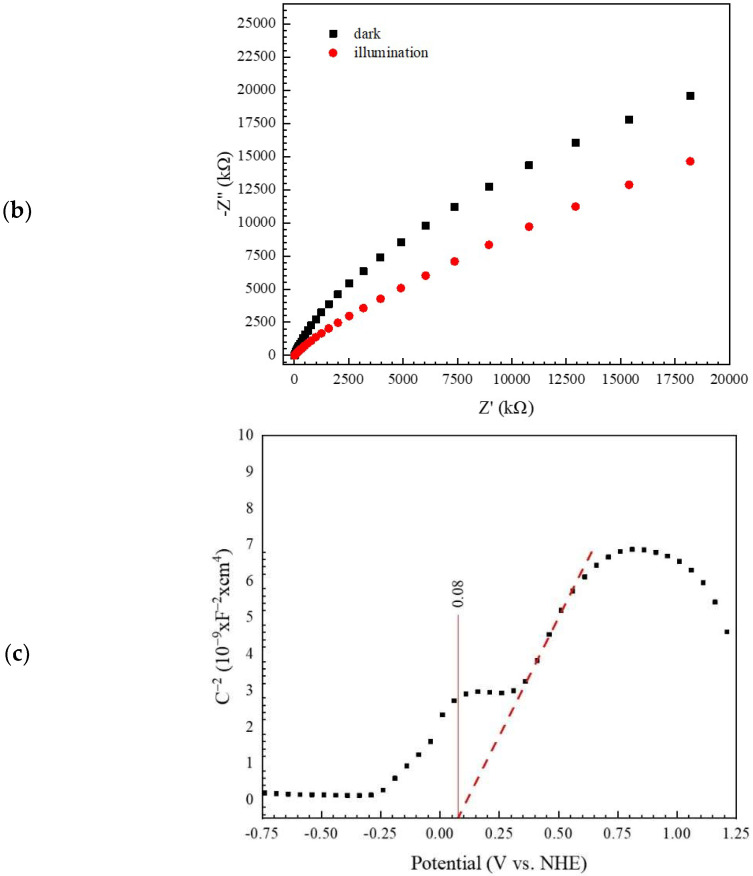
Linear sweep voltammetry scan plots (**a**), electrochemical impedance spectroscopy (EIS) Nyquist plots of the prepared photocatalyst under dark and visible light illumination (**b**), and a Mott-Schottky plot of BiVO_4_ (**c**).

Secondly, the electron-hole separation rate of the catalyst was determined by using electrochemical impedance spectroscopy (EIS) after photo illumination (the visible light from a Panasonic day light lamp, 15W). In principle, the radius of the arc provides information regarding the nature of the charge transfer process at the electrode/electrolyte interface being studied. In general, an increase in radius indicates an enhancement in charge-transfer resistance [36,37,38]. As shown in Figure 6b, the arc radius of the BiVO_4_ catalyst in the dark was larger than that obtained after visible light irradiation, indicating that there were fewer electrons present across the electrolyte interface under the dark condition. On the contrary, a decrease in charge transfer resistance was found after light irradiation. It can be concluded that the PL spectrum, the LSV plots, and the EIS plots confirm the enhanced photoactivity of the synthesized BiVO_4_ catalyst. 

To determine the mechanism of the improved photocatalytic performance of the BiVO_4_ catalyst, the band structure of the prepared catalyst was determined by examining the Mott-Schottky plot (Figure 6c). In principle, the positive slope of the plot can be obtained from the n-type semiconducting photocatalyst and vice versa [3,39]. Determination of the flat band potential (V_FB_) can be carried out by an extrapolation to 1/C^2^ = 0 [40]. Additionally, a calculation of the potential versus Ag/AgCl (known as E_AgCl_) to that versus NHE (called E_NHE_) was carried out using E_NHE_ = E_AgCl_ + 0.210 V. The value of V_FB_ of the prepared BiVO_4_ catalyst was 0.080 eV. It is well accepted that the conduction band potential (V_CB_) of a sample can be approximated from the V_FB_ of the semiconductor [40]. Thus, the sample showed a V_CB_ of about 0.080 eV. By utilizing a band energy of 2.33 eV, the sample showed a valence band potential (V_VB_) of 2.41 eV. 

### 3.2. Photocatalytic Degradation of the Antibiotics

The photocatalytic degradation of the OTC and TC antibiotics was studied under simulated visible light (a Panasonic daylight lamp, 15 W) and natural solar light irradiation.

#### 3.2.1. Photodegradation of OTC Antibiotic 

**Figure 7:**
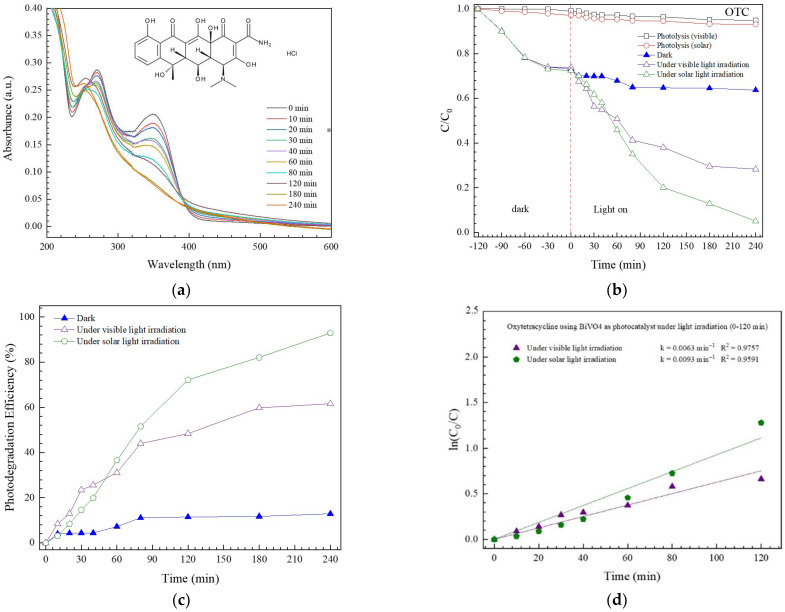
Lowering of absorbance with time toward photodegradation of OTC (**a**), decrease of C/C_0_ with time (**b**), photodegradation efficiency (**c**), and determination of rate constant (**d**).

As seen in Figure 7a, a decrease of absorbance with time was observed, implying the removal of OTC under photo illumination. By monitoring the OTC concentration over time (Figure 7b), the photolysis of this antibiotic was observed to be negligible. Additionally, about 30% of OTC was removed by the adsorption of the antibiotic upon the surface of BiVO_4_. Interestingly, nearly complete degradation of OTC was observed under photo illumination. A degradation performance of about 62% and 93% was observed after illumination of artificial visible light and natural sunlight, respectively (Figure 7c). The photodegradation reaction follows a first-order reaction (Figure 7d). Corresponding rate constants (*k*) of 0.0063 and 0.0093 min^−1^ were detected after visible light and sunlight illumination, respectively. Photocatalytic performance under sunlight was higher than under simulated visible light, supporting the practical use of the synthesized BiVO_4_ catalyst via the utilization of abundant natural sunlight. The removal of toxic antibiotics can be carried out easily and economically using solar energy. 

#### 3.2.2. Photodegradation of TC Antibiotic 

**Figure 8:**
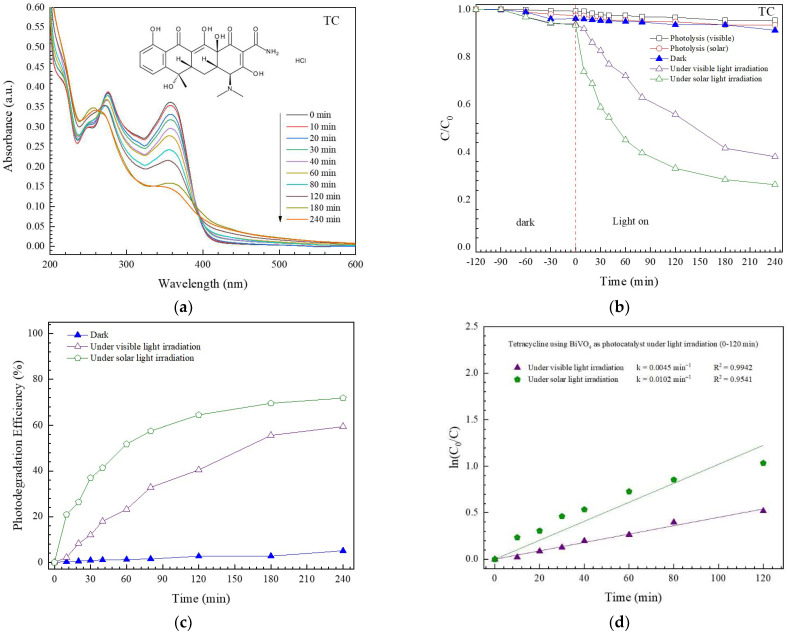
Lowering of absorbance with time toward photodegradation of TC (**a**), a decrease of C/C_0_ with time (**b**), photodegradation efficiency (**c**), and determination of rate constant (**d**).

Figure 8a shows a lowering of TC absorbance with time. This indicates the removal of TC under photoirradiation. As seen in Figure 8b, the removal of TC via photolysis can be neglected. Moreover, lower than 10% of the removal of TC via adsorption processes was observed. The enhanced photocatalytic degradation of TC was observed in the presence of both the photocatalyst and light. Photoactivities of about 59% and 72% were obtained after artificial visible light and natural solar light irradiation, respectively (Figure 8c). The photodegradation reaction correlates well with a first-order reaction (Figure 8d) [11,12,13]. Accordingly, rate constants of 0.0045 and 0.0102 min^−1^ were observed.

#### 3.2.3. Study of Photocatalytic Degradation Mechanism 

**Figure 9:**
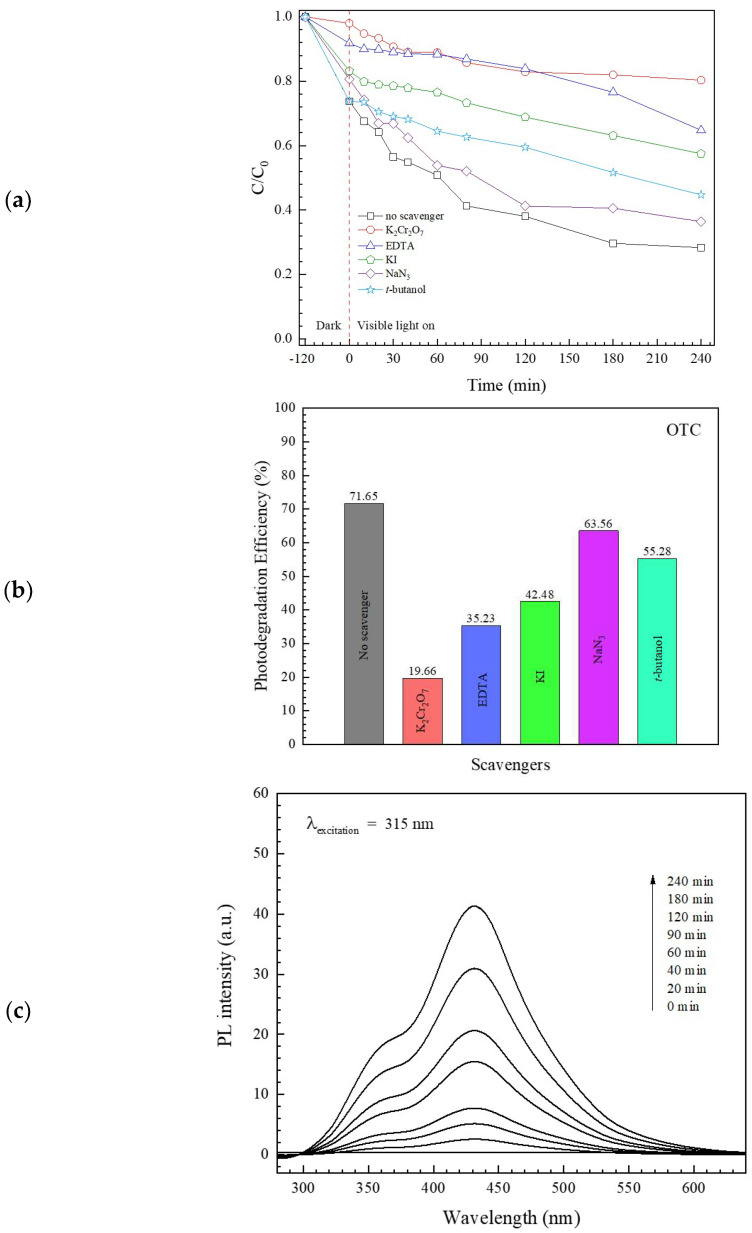
Trapping experiment from photodegradation of OTC (**a**), the corresponding photodegradation efficiency (**b**), and the hydroxyl radical trapping PL spectra of BiVO_4_ after visible light illumination (**c**).

The mechanism for the photocatalytic degradation of OTC antibiotics was determined from the trapping experiment [11,12,13]. The influence of some quenchers on antibiotic removal was elucidated. A dramatic decrease in photocatalytic performance was detected in the presence of K_2_Cr_2_O_7_ and EDTA-2Na (Figure 9a,b), implying the important role of both photogenerated electrons and holes in the degradation of the antibiotic. 

In addition, the determination of hydroxyl radical (•OH) after photo illumination was performed by using a terephthalic acid (TA) probe method [12]. The detection of the fluorescent product 2-hydroxyterephthalic acid (TA-OH) was confirmed from an additional PL peak intensity at 425 nm over time (Figure 9c). The result indicates that the radicals also have a role in the degradation of the antibiotic. 

Firstly, upon photo illumination, the generation of the photogenerated electrons and holes can be found within the conduction band (CB) and the valence band (VB), respectively. Secondly, the generation of the reactive species had occurred. The level of the CB and VB potentials of the prepared BiVO_4_ catalyst can be determined by using Mulliken electronegativity theory [41] as shown:

*E*_VB_ = *χ* − *E*_C_ + 0.5*E*_g_ (4)

*E*_CB_ = *E*_VB_ − *E*_g_ (5)

where *E*_VB_, *E*_CB_ and *E*_C_, and *χ* are the VB, the CB, and the standard hydrogen electrode potential (≈4.5 eV), respectively. *χ* (about 2.34 eV) is the absolute value of the electronegativity of the BiVO_4_ catalyst. The calculated V_VB_ and V_CB_ of the BiVO_4_ catalyst were found to be 2.71 and 0.37 eV, respectively. Practically, however, the CB edge of 0.080 eV was detected from the prepared BiVO4 catalyst (Figure 6c). The band energy of the BiVO_4_ catalyst is 2.33 eV. Thus, the VB edge was found to be 2.41 eV.

In summary, the mechanism regarding the degradation of the antibiotic can be expressed as:

BiVO_4_ + *hν* → BiVO_4_ + *e*^−^ + *h*^+^ (6)

*e*^−^ + O_2_ → •O_2_^−^ (7)

•O_2_^−^+ 2H_2_O + *e*^−^ → 2•OH + 2OH^−^ (8)

*h*^+^ + OH^−^ → •OH (9)

•OH + antibiotic → products(10)

*h*^+^ + antibiotic → products (11)

**Figure 10:**
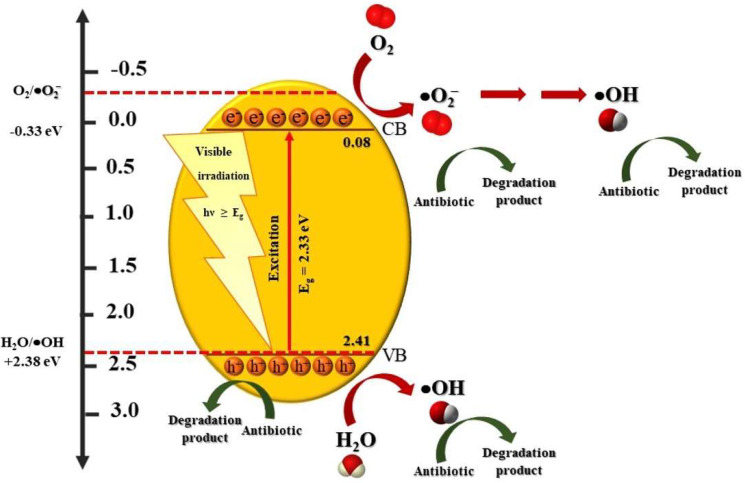
Degradation mechanism of antibiotic by BiVO_4_ photocatalyst.

The details of the photocatalytic degradation mechanism of the toxic antibiotic by the prepared BiVO_4_ catalyst are summarized in Figure 10.

#### 3.2.4. Cycling Ability of the Catalyst

**Figure 11:**
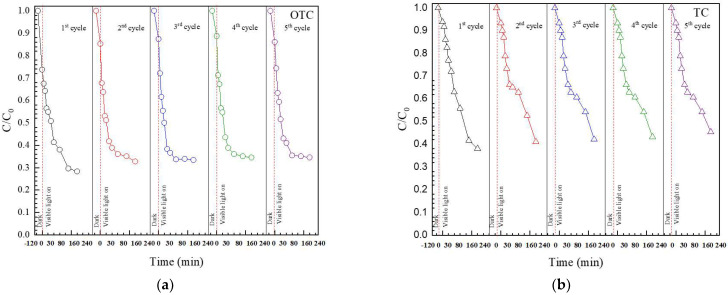
Reusability of the BiVO_4_ catalyst toward degradation of OTC (**a**) and TC (**b**).

**Figure 12:**
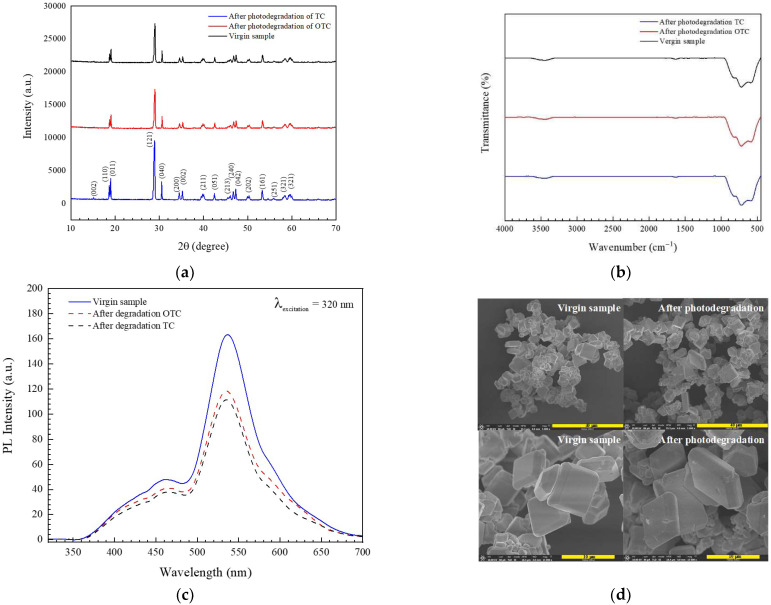
XRD patterns (**a**), FT-IR spectra (**b**), PL spectra (**c**), and SEM micrographs (**d**), of the BiVO_4_ catalyst, before and after the degradation of the antibiotic.

Recycling ability is an important factor affecting the practical application of the prepared photocatalyst [3,11,12,13,14]. Therefore, the reuse of the BiVO_4_ catalyst after the removal of OTC and TC was studied. The synthesized catalyst still showed a promising performance, even after the fifth run (Figure 11). The chemical structure of the catalyst after antibiotic degradation was investigated. The stability of the structure was confirmed (Figure 12a). Identical FT-IR (Figure 12b) and PL spectra (Figure 12c) also evidence the structural stability of the prepared photocatalyst. Furthermore, the morphological structure of the used catalyst is the same as the unused catalyst (Figure 12d) indicating the morphological stability of the sample. 

The photocatalytic efficiency of photocatalysts toward the removal of TC and OTC antibiotics has been previously studied [2,3,4,6,7,8,9,42,43,44,45,46,47,48,49,50,51,52]. In this work, the BiVO_4_ catalyst was used for the degradation of TC and OTC antibiotics under visible light and solar light. The performance of the synthesized BiVO_4_, together with its detected performance from previous works, is summarized in Table 1. On examining OTC degradation, the single component of BiVO_4_ provided a visible-light-responsive activity of 4–83% [3,4,6,42,43,44]. In addition, the BiVO_4_-based binary composites revealed a photoactivity of 68–89% under visible light irradiation [4,6,38,40]. Interestingly, an enhanced efficiency of 90–99% can be obtained by the generation of ternary composites [4,40,41,42]. On examining TC removal, the bare BiVO_4_ showed a low efficiency of 20–60% [2,7,8,44,45,46,47,48,49,50,51,52,53]. The two-component photocatalysts showed an efficiency of 28–92% [2,7,8,9,44,46,47,48,50,51,52,53]. The BiVO_4_-based ternary composites showed an enhanced performance of 68–96% [8,9,44,49,50]. In this research, the prepared BiVO_4_ catalyst exhibited a high photocatalytic performance. About 93% of OTC and 72% of TC can be removed very easily without the doping of metals or the generation of heterostructures. The present finding offers a new photocatalyst with an excellent performance toward the removal of toxic antibiotics in an aqueous solution via the utilization of abundant natural solar energy. 

## 4. Conclusions

A BiVO_4_ photocatalyst was fabricated via a hydrothermal method. The monoclinic photocatalyst showed a band energy of 2.34 eV. Under sunlight conditions, a performance of 72% and 93% degradation of TC and OTC, respectively, was achieved. The photodegradation of the antibiotic followed a first-order reaction. Photogenerated electrons and holes are two major active species concerning the removal of antibiotics. The synthesized BiVO_4_ catalyst still showed a high performance after five times of use, indicating its excellent cycling ability. The presented findings offer a promising route for the creation of a BiVO_4_ catalyst for environmental protection. For future work, the preparation of a floating, or polymer-film, photocatalyst for the degradation of pollutants in wastewater would be worth investigating. This would provide real-scale application of the catalyst in terms of practical work.

## Figures and Tables

**Table 1:** Comparison of oxytetracycline and tetracycline antibiotic degradation via the use of various photocatalysts.

Photocatalyst	Concentration (mg/L)	Catalyst Loading (mg)	Light Source	Lamp	Time (min)	Photodegradation (%)	Ref.
• Photodegradation of oxytetracycline antibiotic							
BiVO_4_	10	50	Visible	15	240	55	[3]
BiVO_4_	10	50	Solar	-	240	83	[3]
BiVO_4_	20	100	Visible	500	70	37	[42]
BiVO_4_	20	50	Visible	1000	120	61	[4]
BiVO_4_	10	50	Visible	350	35	4	[43]
BiVO_4_	10	100	Visible	1000	60	36	[44]
BiVO_4_	10	100	Visible	500	60	47	[6]
BiVO_4_/GO	20	100	Visible	500	70	83	[42]
AgCl/BiVO_4_	20	100	Visible	1000	120	77	[4]
BiVO_4_/TiO_2_	10	100	Visible	1000	60	68	[44]
C/BiVO_4_	10	100	Visible	500	60	89	[6]
Ag/BiVO_4_/GO	20	100	Visible	500	70	90	[42]
Ag/AgCl/BiVO_4_	20	50	Visible	1000	120	98	[4]
BiVO4/TiO2/rGO	10	100	Visible	1000	60	99	[44]
Ag-AgBr/BiVO_4_/Co_3_O_4_	10	50	Visible	350	35	90	[43]
BiVO_4_	10	50	Visible	15	240	62	This work
BiVO_4_	10	50	Solar	-	240	93	This work
• Photodegradation of tetracycline antibiotic							
BiVO_4_	20	50	Visible	300	60	60	[45]
BiVO_4_	10	100	Visible	300	90	56	[46]
BiVO_4_	20	100	Visible	300	80	20	[47]
BiVO_4_	15	200	Visible	32	180	52	[7]
BiVO_4_	20	100	Visible	300	60	41	[8]
BiVO_4_	20	50	Visible	300	240	25	[48]
BiVO_4_	20	100	Visible	300	120	34	[2]
BiVO_4_	30	50	Visible	300	54	54	[49]
BiVO_4_	10	100	Visible	1000	60	34	[44]
BiVO_4_	20	30	Visible	300	25	42	[50]
BiVO_4_	30	50	Visible	500	240	59	[51]
BiVO_4_	20	50	Visible	500	240	20	[52]
ZnO/BiVO_4_	20	50	Visible	300	60	85	[45]
MnFe_2_O_4_/BiVO_4_	10	400	Visible	30	120	92	[53]
Ag/BiVO_4_	20	100	Visible	300	80	65	[47]
Ti/BiVO_4_	15	200	Visible	32	180	60	[7]
g-C_3_N_4_/BiVO_4_	20	100	Visible	300	60	60	[8]
H_2_-BiVO_4_	20	50	Visible	300	240	75	[48]
BiVO_4_/rGH-3	20	100	Visible	300	120	73	[2]
CuO/BiVO_4_	80	100	Visible	300	50	28	[9]
BiVO_4_/TiO_2_	10	100	Visible	1000	60	73	[44]
30%AgI/BiVO_4_	20	30	Visible	300	25	62	[50]
BiVO_4_/ Pal	30	50	Visible	500	240	82	[51]
3%Br/BiVO_4_	20	50	Visible	500	240	79	[52]
Fe_3_O_4_/BiVO_4_/Cds	10	100	Visible	300	90	75	[46]
N-GNDs/BiVO_4_	20	100	Visible	300	80	85	[47]
g-C_3_N_4_/BiVO_4_+PMS	20	100	Visible	300	60	76	[8]
BiVO_4_@PPy/g-C_3_N_4_	30	50	Visible	300	120	90	[49]
CuO/BiVO_4_+PMS	80	50	Visible	300	50	68	[9]
BiVO_4_/TiO_2_/rGO	10	100	Visible	1000	60	96	[44]
30%AgI/rGO/BiVO_4_	20	30	Visible	300	25	84	[50]
BiVO_4_	10	50	Visible	15	240	59	This work
BiVO_4_	10	50	Solar	-	240	72	This work

## Data Availability

Not applicable.

## References

[1] He Q., Xin J., Mao L., Cai X., Ding B., Zhang L., Zhang J., Zheng S., Yang Y. (2021). In situ growth of BiVO_4_/HoVO_4_ heterojunction with OO bond connection for enhanced photodegradation activity. Mater. Lett..

[2] Ma C., Seo W.C., Lee J., Kim Y., Jung H., Yang W. (2021). Chemosphere construction of quantum dots self-decorated BiVO_4_/reduced graphene hydrogel composite photocatalyst with improved photocatalytic performance for antibiotics degradation. Chemosphere.

[3] Senasu T., Youngme S., Hemavibool K., Nanan S. (2021). Sunlight-driven photodegradation of oxytetracycline antibiotic by BiVO_4_ photocatalyst. J. Solid State Chem..

[4] Dai Y., Liu Y., Kong J., Yuan J., Sun C., Xian Q., Yang S., He H. (2019). High photocatalytic degradation efficiency of oxytetracycline hydrochloride over Ag/AgCl/BiVO_4_ plasmonic photocatalyst. Solid State Sci..

[5] Zhang H., Wang Y., Zhai C. (2022). Construction of a novel p-n heterojunction CdS QDs/LaMnO3 composite for photodegradation of oxytetracycline. Mater. Sci. Semicond. Process..

[6] Ye S., Zhou X., Xu Y., Lai W., Yan K., Huang L., Ling J., Zheng L. (2019). Photocatalytic performance of multi-walled carbon nanotube/BiVO_4_ synthesized by electro-spinning process and its degradation mechanisms on oxytetracycline. Chem. Eng. J..

[7] Huyen N.T.K., Pham T.-D., Cam N.T.D., Van Quan P., Van Noi N., Hanh N.T., Tung M.H.T., Dao V.-D. (2021). Fabrication of titanium doped BiVO_4_ as a novel visible light driven photocatalyst for degradation of residual tetracycline pollutant. Ceram. Int..

[8] Kang J., Tang Y., Wang M., Jin C., Liu J., Li S., Li Z., Zhu J. (2021). The enhanced peroxymonosulfate-assisted photocatalytic degradation of tetracycline under visible light by g-C_3_N_4_/Na-BiVO_4_ heterojunction catalyst and its mechanism. J. Environ. Chem. Eng..

[9] Chen X., Zhou J., Chen Y., Zhou Y., Ding L., Liang H., Li X. (2021). Degradation of tetracycline hydrochloride by coupling of photocatalysis and peroxymonosulfate oxidation processes using CuO-BiVO_4_ heterogeneous catalyst. Process Saf. Environ. Prot..

[10] Xu J., Bian Z., Xin X., Chen A., Wang H. (2018). Size dependence of nanosheet BiVO_4_ with oxygen vacancies and exposed {001} facets on the photodegradation of oxytetracycline. Chem. Eng. J..

[11] Chankhanittha T., Yenjai C., Nanan S. (2022). Utilization of formononetin and pinocembrin from stem extract of *Dalbergia parviflora* as capping agents for preparation of ZnO photocatalysts for degradation of RR141 azo dye and ofloxacin antibiotic. Catal. Today.

[12] Chankhanittha T., Komchoo N., Senasu T., Piriyanon J., Youngme S., Hemavibool K., Nanan S. (2021). Silver decorated ZnO photocatalyst for effective removal of reactive red azo dye and ofloxacin antibiotic under solar light irradiation. Colloids Surf. A Physicochem. Eng. Asp..

[13] Sansenya T., Masri N., Chankhanittha T., Senasu T., Piriyanon J., Mukdasai S., Nanan S. (2022). Hydrothermal synthesis of ZnO photocatalyst for detoxification of anionic azo dyes and antibiotic. J. Phys. Chem. Solids.

[14] Nur A.S., Sultana M., Mondal A., Islam S., Robel F.N., Islam A., Sumi M.S.A. (2022). A review on the development of elemental and codoped TiO_2_ photocatalysts for enhanced dye degradation under UV–vis irradiation. J. Water Process Eng..

[15] Belousov A.S., Suleimanov E.V. (2021). Application of metal–organic frameworks as an alternative to metal oxide-based photocatalysts for the production of industrially important organic chemicals. Green Chem..

[16] Subhiksha V., Kokilavani S., Khan S.S. (2022). Recent advances in degradation of organic pollutant in aqueous solutions using bismuth based photocatalysts: A review. Chemosphere.

[17] Belousov A.S., Suleimanov E.V., Fukina D.G. (2021). Pyrochlore oxides as visible light-responsive photocatalysts. New J. Chem..

[18] Abideen Z.U., Teng F., Gu W., Yang Z., Zhang A., Zhao F., Shah A.H. (2020). Enhanced visible light photocatalytic activity of CeO_2_@Zn0.5Cd0.5S by facile Ce(IV)/Ce(III) cycle. Arab. J. Chem..

[19] Abideen Z.U., Teng F. (2020). Fe_2_O_3_-promoted interface charge separation and visible-light activity of Fe_2_O_3_@Zn0.3Cd0.7S. Mater. Chem. Phys..

[20] Shah A.H., Gu W., Abideen Z.U., Teng F. (2020). Removal of chromium from aqueous solution by porous Bi_2_MoO_6_@BiOCl nanostructure. J. Solid State Chem..

[21] Gu W., Teng F., Chu Y., Zhang A., Abideen Z.U. (2019). An interesting charge accumulation process of Bi_12_O_15_Cl_6_. J. Electroanal. Chem..

[22] Gu W., Xu J., Teng F., Abideen Z.U. (2018). Investigation on the Different Photocatalytic Properties of Bismuths Oxychlorides: Bi_12_O_15_Cl_6_ versus Bi_3_O_4_Cl versus BiOCl. ChemistrySelect.

[23] Abideen Z.U., Teng F. (2018). Enhanced photochemical activity and stability of ZnS by a simple alkaline treatment approach. CrystEngComm.

[24] Chankhanittha T., Somaudon V., Watcharakitti J., Piyavarakorn V., Nanan S. (2020). Performance of solvothermally grown Bi_2_MoO_6_ photocatalyst toward degradation of organic azo dyes and fluoroquinolone antibiotics. Mater. Lett..

[25] Rathi V., Panneerselvam A., Sathiyapriya R. (2020). A novel hydrothermal induced BiVO_4_/g-C_3_N_4_ heterojunctions visible-light photocatalyst for effective elimination of aqueous organic pollutants. Vacuum.

[26] Senasu T., Narenuch T., Wannakam K., Chankhanittha T., Nanan S. (2020). Solvothermally grown BiOCl catalyst for photodegradation of cationic dye and fluoroquinolone-based antibiotics. J. Mater. Sci. Mater. Electron..

[27] Peleyeju G.M., Umukoro E.H., Babalola J.O., Arotiba O.A. (2020). Solar-Light-Responsive Titanium-Sheet-Based Carbon Nanoparticles/B-BiVO_4_/WO_3_ Photoanode for the Photoelectrocatalytic Degradation of Orange II Dye Water Pollutant. ACS Omega.

[28] Chen S.-H., Jiang Y.-S., Lin H.-Y. (2020). Easy Synthesis of BiVO_4_ for Photocatalytic Overall Water Splitting. ACS Omega.

[29] Baral B., Parida K. (2020). {040/110} Facet Isotype Heterojunctions with Monoclinic Scheelite BiVO_4_. Inorg. Chem..

[30] Mudavakkat V., Atuchin V., Kruchinin V., Kayani A., Ramana C. (2012). Structure, morphology and optical properties of nanocrystalline yttrium oxide (Y_2_O_3_) thin films. Opt. Mater..

[31] Ji H., Huang Z., Xia Z., Molokeev M.S., Jiang X., Lin Z., Atuchin V.V. (2015). Comparative investigations of the crystal structure and photoluminescence property of eulytite-type Ba_3_Eu(PO_4_)_3_and Sr_3_Eu(PO_4_)_3_. Dalton Trans..

[32] Atuchin V., Chimitova O., Adichtchev S., Bazarov J., Gavrilova T., Molokeev M., Surovtsev N., Bazarova Z. (2013). Synthesis, structural and vibrational properties of microcrystalline β-RbSm(MoO_4_)_2_. Mater. Lett..

[33] Wang G.-L., Shan L.-W., Wu Z., Dong L.-M. (2017). Enhanced photocatalytic properties of molybdenum-doped BiVO_4_ prepared by sol–gel method. Rare Met..

[34] Zhang Z., Wang M., Cui W., Sui H. (2017). Synthesis and characterization of a core–shell BiVO_4_@g-C_3_N_4_ photo-catalyst with enhanced photocatalytic activity under visible light irradiation. RSC Adv..

[35] Yanga R., Zhua Z., Hua C., Zhongb S., Zhangb L., Liuad B., Wangc W. (2020). One-step preparation (3D/2D/2D) BiVO_4_/FeVO_4_@rGO heterojunction composite photocatalyst for the removal of tetracycline and hexavalent chromium ions in water. Chem. Eng. J..

[36] Ma J., Jin D., Li Y., Xiao D., Jiao G., Liu Q., Guo Y., Xiao L., Chen X., Li X. (2021). Photocatalytic conversion of biomass-based monosaccharides to lactic acid by ultrathin porous oxygen doped carbon nitride. Appl. Catal. B Environ..

[37] Jiang W., Li Z., Liu C., Wang D., Yan G., Liu B., Che G. (2021). Enhanced visible-light-induced photocatalytic degradation of tetracycline using BiOI/MIL-125(Ti) composite photocatalyst. J. Alloy. Compd..

[38] Ma F., Yang Q., Wang Z., Liu Y., Xin J., Zhang J., Hao Y., Li L. (2018). Enhanced visible-light photocatalytic activity and photostability of Ag_3_PO_4_/Bi_2_WO_6_ heterostructures toward organic pollutant degradation and plasmonic Z-scheme mechanism. RSC Adv..

[39] Meng Q., Zhang B., Fan L., Liu H., Valvo M., Edström K., Cuartero M., de Marco R., Crespo G.A., Sun L. (2019). Efficient BiVO_4_ Photoanodes by Postsynthetic Treatment: Remarkable Improvements in Photoelectrochemical Performance from Facile Borate Modification. Angew. Chem. Int. Ed..

[40] Wang T., Bai Y., Si W., Mao W., Gao Y., Liu S. (2021). Heterogeneous photo-Fenton system of novel ternary Bi_2_WO_6_/BiFeO_3_/g-C_3_N_4_ heterojunctions for highly efficient degrading persistent organic pollutants in wastewater. J. Photochem. Photobiol. A Chem..

[41] Ni S., Zhou T., Zhang H., Cao Y., Yang P. (2018). BiOI/BiVO_4_ Two-Dimensional Heteronanostructures for Visible-Light Photocatalytic Degradation of Rhodamine B. ACS Appl. Nano Mater..

[42] Ouyang K., Yang C., Xu B., Wang H., Xie S. (2021). Synthesis of novel ternary Ag/BiVO_4_/GO photocatalyst for degradation of oxytetracycline hydrochloride under visible light. Colloids Surf. A Physicochem. Eng. Asp..

[43] Chen F., Wu C., Wang J., François-Xavier C.P., Wintgens T. (2019). Highly efficient Z-scheme structured visible-light photocatalyst constructed by selective doping of Ag@AgBr and Co_3_O_4_ separately on {010} and {110} facets of BiVO_4_: Pre-separation channel and hole-sink effects. Appl. Catal. B Environ..

[44] Wang W., Han Q., Zhu Z., Zhang L., Zhong S., Liu B. (2019). Enhanced photocatalytic degradation performance of organic contaminants by heterojunction photocatalyst BiVO_4_/TiO_2_/RGO and its compatibility on four different tetracycline antibiotics. Adv. Powder Technol..

[45] Li Y., Sun X., Tang Y., Ng Y.H., Li L., Jiang F., Wang J., Chen W., Li L. (2021). Understanding photoelectrocatalytic degradation of tetracycline over three-dimensional coral-like ZnO/BiVO_4_ nanocomposite. Mater. Chem. Phys..

[46] Xu G., Du M., Li T., Guan Y., Guo C. (2021). Facile synthesis of magnetically retrievable Fe_3_O_4_/BiVO_4_/CdS heterojunction composite for enhanced photocatalytic degradation of tetracycline under visible light. Sep. Purif. Technol..

[47] Ma C., Din S.T.U., Seo W.C., Lee J., Kim Y., Jung H., Yang W. (2021). BiVO_4_ ternary photocatalyst co-modified with N-doped graphene nanodots and Ag nanoparticles for improved photocatalytic oxidation: A significant enhancement in photoinduced carrier separation and broad-spectrum light absorption. Sep. Purif. Technol..

[48] Yang C., Qin C., Zhong J., Li J., Huang S., Wang Q., Ma L. (2021). Photocatalytic enhancement mechanism insight for BiVO_4_ induced by plasma treatment under different atmospheres. J. Alloy. Compd..

[49] Yan L., Li W., Zhao Q., Zhu Z., Hu C., Liu B. (2021). Enhanced photocatalytic conversion of (3D/2D) BiVO_4_@Polypyrrole/g-C_3_N_4_ ternary composites with Z-scheme band alignment for the Antibiotic removal. Colloids Surf. A Physicochem. Eng. Asp..

[50] Lakhera S.K., Hafeez H.Y., Venkataramana R., Veluswamy P., Choi H., Neppolian B. (2019). Design of a highly efficient ternary AgI/rGO/BiVO_4_ nanocomposite and its direct solar light induced photocatalytic activity. Appl. Surf. Sci..

[51] Shi Y., Hu Y., Zhang L., Yang Z., Zhang Q., Cui H., Zhu X., Wang J., Chen J., Wang K. (2017). Palygorskite supported BiVO_4_ photocatalyst for tetracycline hydrochloride removal. Appl. Clay Sci..

[52] Qin C., Liao H., Rao F., Zhong J., Li J. (2020). One-pot hydrothermal preparation of Br-doped BiVO_4_ with enhanced visible-light photocatalytic activity. Solid State Sci..

[53] Cam N.T.D., Pham H.D., Pham T.D., Phuong T.T.T., van Hoang C., Tung M.H.T., Trung N.T., Huong N.T., Hien T.T.T. (2021). Novel photocatalytic performance of magnetically recoverable MnFe_2_O_4_/BiVO_4_ for polluted antibiotics degradation. Ceram. Int..

